# IGFBP-1 in Cardiometabolic Pathophysiology—Insights From Loss-of-Function and Gain-of-Function Studies in Male Mice

**DOI:** 10.1210/jendso/bvz006

**Published:** 2019-11-04

**Authors:** Natalie J Haywood, Thomas A Slater, Michael Drozd, Nele Warmke, Connor Matthews, Paul A Cordell, Jessica Smith, Jethro Rainford, Harneet Cheema, Caitlyn Maher, Katherine I Bridge, Nadira Y Yuldasheva, Richard M Cubbon, Mark T Kearney, Stephen B Wheatcroft

**Affiliations:** Leeds Institute of Cardiovascular and Metabolic Medicine, Faculty of Medicine and Health, University of Leeds, Leeds, UK

**Keywords:** insulin-like growth factor binding protein-1, endothelial repair, inflammatory monocytes, reparative monocytes

## Abstract

We have previously reported that overexpression of human insulin-like growth factor binding protein (IGFBP)-1 in mice leads to vascular insulin sensitization, increased nitric oxide bioavailability, reduced atherosclerosis, and enhanced vascular repair, and in the setting of obesity improves glucose tolerance. Human studies suggest that low levels of IGFBP-1 are permissive for the development of diabetes and cardiovascular disease. Here we seek to determine whether loss of IGFBP-1 plays a causal role in the predisposition to cardiometabolic disease. Metabolic phenotyping was performed in transgenic mice with homozygous knockout of IGFBP-1. This included glucose, insulin, and insulin-like growth factor I tolerance testing under normal diet and high-fat feeding conditions. Vascular phenotyping was then performed in the same mice using vasomotor aortic ring studies, flow cytometry, vascular wire injury, and angiogenesis assays. These were complemented with vascular phenotyping of IGFBP-1 overexpressing mice. Metabolic phenotype was similar in IGFBP-1 knockout and wild-type mice subjected to obesity. Deletion of IGFBP-1 inhibited endothelial regeneration following injury, suggesting that IGFBP-1 is required for effective vascular repair. Developmental angiogenesis was unaltered by deletion or overexpression of IGFBP-1. Recovery of perfusion following hind limb ischemia was unchanged in mice lacking or overexpressing IGFBP-1; however, overexpression of IGFBP-1 stimulated hindlimb perfusion and angiogenesis in insulin-resistant mice. These findings provide new insights into the role of IGFBP-1 in metabolic and vascular pathophysiology. Irrespective of whether loss of IGFBP-1 plays a causal role in the development of cardiometabolic disorders, increasing IGFBP-1 levels appears effective in promoting neovascularization in response to ischemia.

The hormone insulin and related peptides insulin-like growth factors (IGFs) have diverse actions in mammalian physiology. The family of insulin-like growth factor binding proteins (IGFBPs) bind IGFs with high affinity and confer temporal and spatial regulation to IGF bioactivity [1]. Several members of the IGFBP family also exert cellular effects independent of IGF binding and have been identified as putative signaling molecules implicated in a range of physiological processes [2]. Recently, important roles of other members of the IGF axis, particularly the IGF binding proteins, have become apparent in obesity, diabetes, and cardiovascular disease [3].

IGFBP-1 is a 30-kDa protein, derived mainly from the liver. Under normal conditions, changes in plasma insulin concentrations form the primary mechanisms by which IGFBP-1 levels are regulated via insulin response elements in the IGFBP-1 promoter region, which confers insulin inhibition of IGFBP-1 expression [4, 5]. There is a negative correlation between IGFBP-1 and biomarkers of cardiovascular disease such as blood pressure, body mass index (BMI), waist/hip ratio, and fasting insulin levels [6–8], and in prospective studies reduced IGFBP-1 levels strongly predict the long-term development of diabetes [9–11] and of fatal coronary heart disease independently of other risk factors [12]. Cross-sectional data also support an association between low IGFBP-1 levels, cardiovascular risk, and subclinical atherosclerosis [6, 13, 14]. Collectively, these data suggest that low levels of IGFBP-1 are permissive for the development of both diabetes and cardiovascular disease.

We have previously reported that human IGFBP-1, when overexpressed in mice, leads to vascular insulin sensitization; increased nitric bioavailability; reduced atherosclerosis [15]; and enhanced vascular repair [16]. In the setting of obesity, we found that IGFBP-1 enhanced whole-body glucose tolerance and insulin sensitivity through interaction of its RGD domain with cell surface integrin receptors [17]. Although these overexpression studies provide persuasive evidence for a putative therapeutic role of IGFBP-1, they are unable to delineate the role of IGFBP-1 in normal physiology. To examine whether the presence of IGFBP-1 is obligatory to maintain metabolic and vascular homeostasis, it is necessary to employ a loss-of-function approach in mice with deletion of IGFBP-1.

Several members of the IGFBP family have now been ascribed modulatory roles in vascular endothelial pathophysiology [18]. By influencing functional properties of endothelial cells, IGFBPs may confer either stimulatory or inhibitory influences on angiogenesis [19]. Although these effects are well reported for IGFBP-2, -3, -4, -5, and -6, the potential influence of IGFBP-1 on angiogenesis has not been studied.

In this report we undertook metabolic and vascular phenotyping in IGFBP-1-knockout (IGFBP-1-KO) mice to determine whether IGFBP-1 is an obligatory player in normal physiology. We also examined the effects of IGFBP-1 on developmental and ischemia-driven angiogenesis in IGFBP-1-KO and IGFBP-1-overexpressing mice.

## Material and Methods

### Animal husbandry

IGFBP-1-KO mice [20–22] (Jackson Laboratory Stock Id 005248) and IGFBP-1-transgenic (IGFBP-1-tg) mice (Jackson Laboratory Stock Id 008221) were purchased from the Jackson Laboratory and the colonies maintained at the University of Leeds. IRKO mice hemizygous for knockout of the insulin receptor were originally obtained from the Mouse Mammalian Genetics Unit at MRC Harwell, UK [21, 22]. IRKO × IGFBP-1 mice were generated by crossing IGFBP-tg males with IRKO females. The protocol was approved by the University of Leeds Ethics Committee (A275) and all experiments were carried out under the authority of UK Home Office Licenses PPL 70/8115 and P144DD0D6 and were performed on male mice only from the age of 8 weeks, unless otherwise stated. Cages were maintained in humidity- (55%) and temperature-controlled (22°C) conditions with a 12-hour light/dark cycle. To induce obesity, 8 week old male mice were fed a high-fat diet for 8 weeks (Research Diets, D12492). Mice were genotyped by Transnetyx (Cordova, TN).

### In vivo metabolic studies

Metabolic profiling was performed using glucose, insulin, and IGF-I tolerance tests. Mice were fasted for 16 hours prior to the glucose tolerance test or 2 hours prior to insulin and IGF-I tolerance tests. Blood glucose was measured using a hand-held Glucose Meter (Accu-Chek Aviva) taken from the tail vein. An intraperitoneal injection of glucose (1 mg/g), insulin (Actrapid; Novo Nordisk, Bagsvaerd, Denmark) (0.75 IU/kg) or IGF-I (Gropep) (0.75 µg/g) was performed. Glucose concentration was measured at 30-minute intervals for 2 hours from the point of glucose/insulin/IGF-I administration. Mice were not restrained between measurements.

### Histological assessment of liver

Samples for histology were fixed in 4% paraformaldehyde (PFA) for at least 24 hours, before processing in ethanol and embedding in paraffin. Five micrometer sections were taken. After drying, slides were stained with hematoxylin and eosin.

For assessment of nonalcoholic fatty liver disease (NAFLD) in sections of murine liver, a validated rodent NAFLD scoring system was used [23], which takes into account micro and macrosteatosis, inflammation, and hypertrophy. Each sample was assessed by at least two independent verifiers and the average score per sample taken.

### Arterial injury

A small incision was made in the midthigh to permit isolation of the femoral artery. Following an arteriotomy made using iris scissors (World-Precision Instruments, Sarasota, FL), a 0.014-inch-diameter angioplasty guide wire with tapered tip (Hi-torque Cross-it XT, Abbott-Vascular, Abbott, IL) was introduced. The angioplasty guide wire was advanced 3 cm, and 3 passages were performed per mouse, resulting in complete arterial denudation as demonstrated in pilot studies in vessels harvested at day 0. The guide wire was removed and the suture was tightened rapidly. The vessel was then ligated, and the skin was closed with a continuous suture. The contralateral artery underwent an identical sham operation, without passage of the wire. Animals received postoperative analgesia with buprenorphine (0.25 mg/kg).

Mice were anesthetized 5 days after wire injury, and 50 μL of 0.5% Evans blue dye injected into the inferior vena cava. The mice were perfused/fixed with 4% paraformaldehyde in phosphate-buffered saline (PBS) before the femoral arteries (injured and uninjured) were harvested. The vessels were opened longitudinally. The areas stained and unstained in blue were measured in a 5-mm injured segment beginning 5 mm distal to the aortic bifurcation, and the percentage areas were calculated using ImageProPlus7.0 software (Media Cybernetics, Bethesda, MD) [16, 24, 25].

### Vascular function

Vasomotor function was assessed in aortic rings of 12-week-old mice, as previously described [26]. The thoracic aorta was harvested and divided into 5-mm rings. These rings were mounted in separate chambers of an organ bath containing Krebs–Henseleit buffer (composition in mmol/L: NaCl 119, KCl 4.7, KH_2_PO_4_ 1.18, NaHCO_3_25, MgSO_4_ 1.19, CaCl_2_ 2.5, and glucose 11.0) and perfused with 95% O_2_/5% CO_2_. Passive tension was increased gradually to 3 g and equilibrated to stabilize rings before commencing experiments. Relaxation responses to cumulative addition of acetylcholine (1 nmol/L to 100 μmol/L) were first assessed after preconstriction with 300 nmol/L of the vasoconstrictor phenylephrine (PE). A cumulative dose–response to PE (1 nmol/L to 100 μmol/L) was then performed. Finally, the relaxation response to sodium nitroprusside (SNP) (0.01 nmol/L to 100 μmol/L) was assessed to examine endothelium-independent vasodilation. Relaxation responses are expressed as percentage decrement in preconstricted tension. Chambers were washed with Krebs–Henseleit buffer and rings equilibrated back to 3 g of tension between additions of each substrate.

### Flow cytometry

Whole blood underwent red blood cell lysis (BD PharmLyseTM). Cells were washed and resuspended in PBS containing 0.5% bovine serum albumin (BSA) (Sigma-Aldrich) and 2 mM ethylenediamine tetra-acetate (EDTA) (Sigma-Aldrich). Fc receptors were blocked with a CD16/32 FcR Blocking Reagent (Miltenyi Biotec, 130-092-575) for 10 minutes at 4°C. Samples were then incubated with anti-CD45-VioBlue (Miltenyi Biotec, 130-110-802) [27], anti-CD11b-FITC (Miltenyi Biotec, 130-081-201) [28], anti-Ly6G-PE (Miltenyi Biotec, 130-107-913) [29], and anti-Ly6C-APC (eBioscience, 17-5932-82) [30] for 10 minutes at 4°C, according to the manufacturer’s protocol. Stained cells were washed in PBS containing 0.5% BSA and 2 mM EDTA. Samples were analyzed by flow cytometry (CytoFLEX S, Beckman Coulter). Leukocytes were identified based on typical light scatter properties, with further gating to define CD45^+^ leukocytes, CD45^+^CD11b^+^ myeloid cells, CD45^+^CD11b^+^Ly6G^-^Ly6C^hi^ inflammatory monocytes, CD45^+^CD11b^+^Ly6G^-^Ly6C^low^ reparative monocytes, CD45^+^CD11b^+^Ly6G^hi^Ly6C^hi^ neutrophils. Data were scaled to cells/mL of blood.

### Retinal angiogenesis

To determine the effect of IGFBP-1 on developmental angiogenesis, a retinal angiogenesis assay was used, as previously described [31]. Briefly, IGFBP-1-KO and IGFBP-1-tg and their respective wild-type littermate control P5 male and female pups were euthanized, eyes harvested, and eye balls were fixed for 2 hours at room temperature in 4% PFA and left in PBS at 4°C until the retinas were dissected. Retinas were stained using Isolectin B4-Alexa-488 (Thermo Scientific, I21411), mounted with ProLong Gold (Thermo Scientific, P10144), and imaged using an LSM700 confocal microscope.

All image analysis was performed blinded by 2 independent individuals; averages were taken of both results. The angiogenic criteria were radial outgrowth defined as distance from the outer ring of the optical disk to the vascular front, taking a mean of 3 measurements per segment of each retina; branching points (in a 200 µm × 200 µm square) in 6 to 8 areas spread across the retina and the mean calculated; vessel density % of the vasculature in the peripheral part of the retina; tip cells defined as the number/100 µm of the vascular front and the mean calculated over 3 areas per image, and filopodia defined as the number/tip cell) from 2/3 tip cells per image and the mean calculated.

### Hindlimb ischemia

Hindlimb ischemia was used as an experimental model of pathological angiogenesis, as previously described [32]. Briefly, mice were anesthetized with isoflurane (2.5–5%) before the left femoral artery was exposed, dissected free, ligated proximally and distally, and excised. Animals received postoperative analgesia with buprenorphine (0.25 mg kg^–1^). Hindlimb blood flow was quantified using a laser Doppler blood flow analyzer (Moor instruments) on postoperative days 0, 7, 14, and 21. Hindlimb blood flow is expressed as a percentage of ischemic to nonischemic limb blood flow.

Day 7 after surgery, gastrocnemius from both ischemic and nonischemic limbs was harvested under terminal anesthesia and fixed in 4% PFA for 1 hour at room temperature. Muscles were then embedded in Optimal Cutting Temperature compound (OCT) (Cellpath, KMA-0100-00A) and stored at –80°C until sectioned. Sections (10 µM) were taken using a Leica CM3050 S Research Cryostat.

Slides were blocked and permeabilized in PBS + 0.25% Triton-X100 + 1% BSA + for 1 hour. Then stained with Isolectin B4-Alexa Fluor-488 (Invitrogen I21411) at 1/100 in PBS + 0.25% Triton + 1% BSA for 1 hour. Slides were washed 3 times in PBS and mounted with a coverslip using Prolong Gold with DAPI (P36931, ThermoFisher). Slides were imaged using an LSM700 confocal microscope.

### Plasma samples

Blood samples were collected from the lateral saphenous vein (EDTA collection tubes Sarstedt 16.444). Samples were then spun at 10,000 RPM for 10 minutes in a bench top centrifuge. Plasma was stored at –20°C until used. IGFBP-1 (Abcam ab213865), IGFBP-2 (Abcam ab207615), IGFBP-3 (R&D Systems, MGB300), insulin (Crystal Chem, 90080), and IGF-I (R&D Systems MG100) levels were measured as per kit instructions.

### Protein expression

Tissue was harvested under terminal anesthesia and snap frozen. Tissue was homogenized and lysed in cell extraction buffer (FNN0011, Invitrogen, Carlsbad, CA) and protein content was quantified by a bicinchoninic acid assay (BCA) assay (Sigma-Aldrich, St. Louis, MO). Fifty micrograms of protein were resolved on a 4% to 12% Bis-Tris gel (Bio-Rad, Hemel Hempstead, UK) and transferred to nitrocellulose membranes. Membranes were probed with antibodies diluted in 5% BSA; 1:1000 AKT (cell signaling, #9272) [33], 1:1000 MAPK (Cell signaling, #4695) [34], and 1:20000 beta actin (Sc-47778; Santa Cruz) [35], before incubation with appropriate secondary horseradish peroxidase-conjugated antibody [36, 37]. Blots were visualized with Immobilon Western Chemiluminescence HRP Substrate (Merck Millipore, Watford, UK) and imaged with Syngene chemiluminescence imaging system (SynGene, Cambridge, UK).

### Recombinant full length human IGFBP-1 expression

Recombinant IGFBP-1 used in in vitro experiments was produced as previously described [17]. Briefly, the expression vector containing the DNA coding sequence of the mature human IGFBP-1 polypeptide was amplified from Image clone 4800940 and subcloned into pM-secSUMOstar vector. A mutant IGFBP-1 protein, unable to bind integrins, was created by altering the IGFBP-1 “RGD” motif sequence to WGD [38] using the Quikchange system (Agilent). An amino-terminal truncated coding sequence of IGFBP-1 lacking the IGF-I binding domain [39] was amplified by PCR from the IGFBP-1 cDNA clone using primers 5′-ggggactcacgtctcgaggtctgccgggggagca-3′ (f) and 5′- gacagaacattatttcatctagattcagttttgtac-3′ (r) and subcloned into pM-secSUMOstar vector as described for the full-length coding sequence. The SUMOstar coding sequence was fused to the amino terminus of truncated IGFBP-1 sequence (Leu 87 to Asn259 of the preprotein with a GlyGly linker between the 2 sequences).

For protein expression, Expi293F cells (Life Technologies) were transiently transfected with the expression vector using Expifectamine as detailed by the manufacturer’s instructions, with medium harvested 7 days after transfection. After removal of cells and cell debris by centrifugation (10 minutes at 300*g* then 10 minutes at 4500*g*) and addition of protease inhibitor cocktail and phosphatase inhibitor cocktail 3 (both from Sigma), medium was passed through a 0.2-µm filter and protein was precipitated by addition of 2 volumes of saturated ammonium sulphate at 4°C followed by incubation on ice for 1 hour. After centrifugation (4500*g* at 4°C for 1 hour) floating protein pellets were redissolved in Dulbecco’s PBS (DPBS) and residual ammonium sulphate was removed by gel filtration with DPBS-equilibrated Zeba gel filtration spin columns (Fisher Scientific). His_6_SUMO–IGFBP-1 fusion protein was then isolated using HisPur Cobalt spin columns (Fisher Scientific) as directed by the manufacturer’s instructions. Eluates were buffer-exchanged to DPBS using Zeba columns prior to digestion of His_6_SUMO–IGFBP-1 with SUMOstar protease. Cleaved His_6_SUMOstar was removed with HisPur Cobalt columns and eluant containing IGFBP-1 was then applied to a Sephacryl S100 column equilibrated with DPBS at room temperature using an Akta Avant chromatography system (GE Healthcare). Purity was confirmed to ≥95% by Coomassie staining of sodium dodecyl sulfate polyacrylamide gel electrophoresis (SDS-PAGE) gels. N-del mutant SUMO-IGFBP-1 was resistant to cleavage by SumoSTAR protease and was used as the intact fusion protein in experiments.

### Tube formation

Human umbilical vein endothelial cells (HUVECs) (Promocell, C-12203) were pre-treated for 24 hours with 500 ng/mL IGFBP-1 or PBS control or IGF-I neutralizing antibody (R and D systems AF-291-NA). Cells were washed once with PBS, trypsinized (Thermo fisher Scientific, 12604013) and resuspended in growth media (M199 (sigma, M4530), 20% FCS, 20 mM HEPES, 1% AAS (Thermo fisher Scientific, 15240062), 15 µg/mL ECGS (Sigma, E2759), 2 mM Sodium Pyruvate, 5 U/mL heparin) and seeded at 100 000 cells per well of a Matrigel-coated (Beckton Dickinson, 734-0268) 24-well plate and incubated for 4 hours at 37°C. Endothelial tube formation was evaluated as the mean number of tubes formed per high-power field (HPF) (×40).

### Proliferation

HUVECS were seeded at 25,000 cells per well of a 24-well plate and left to settle overnight. HUVECs were pretreated for 1 hour with 500 ng/mL IGFBP-1 or PBS control and then used in a fluorescent EdU proliferation assay, as per kit instruction (Thermo fisher Scientific, C10337).

### Cytodex bead

HUVECs were mixed with Cytodex 3 microcarriers (Amersham 17-0485-01) at a concentration of 400 HUVECs per bead in 1 mL of growth media. Beads with cells were shaken gently every 20 minutes for 4 hours at 37°C and 5% CO_2_. After incubating, beads with cells were transferred to a 25-cm^2^ tissue culture flask and left overnight in 5 mL of media supplemented with rIGFBP-1 (500 ng/mL) at 37°C and 5% CO_2_. The following day, beads with cells were washed 3 times with 1 mL of media and resuspended at a concentration of 200 cell-coated beads/mL in 2 mg/mL of fibrinogen (Sigma-Aldrich F-8630) with 0.15 units/mL of aprotinin (Sigma-Aldrich A-1153), 5 ng/mL VEGF, and 5 ng/mL FGF. A total of 500 µL of fibrinogen/bead solution was added to 0.625 units of thrombin (Sigma-Aldrich T-3399) in 1 well of a 24-well tissue culture plate. Fibrinogen/bead solution was allowed to clot for 5 minutes at room temperature and then at 37°C and 5% CO_2_ for 20 minutes. One milliliter of media supplemented with rIGFBP-1 (500 ng/mL) was then added and incubation continued at 37°C and 5% CO_2_. Beads were imaged 24 hours later [40].

### IGF-I ligand blot

The ability of rIGFBP-1 and mutant to bind IGF-I was assessed by ligand blotting using biotinylated IGF-I [41]. Purified recombinant IGF-I (R&D systems) was biotinylated with EZ-Link™ Sulfo-NHS-Biotin (Thermo) according to the manufacturer’s protocol. Protein samples containing IGFBP-1 were resolved by nonreducing SDS-PAGE then electrotransferred to PVDF membranes, blocked with 5% BSA in tris buffered saline (TBS)-T and probed with biotinylated IGF-I in TBS-T/5% BSA followed by Streptavidin-HRP (Cell Signalling Technologies) and chemiluminescent imaging was performed as described for western blot experiments.

### Data analysis

All data are shown as the mean ± standard error of the mean (SEM). All image analysis was performed in ImageJ. Student unpaired t-test or analysis of variance were used where appropriate and performed with GraphPad Prism software version 7.

### Data availability

The datasets generated during and/or analyzed during the current study are not publicly available but are available from the corresponding author on reasonable request.

## Results

### Metabolic phenotype of IGFBP-1 knockout mice

**Figure 1.**
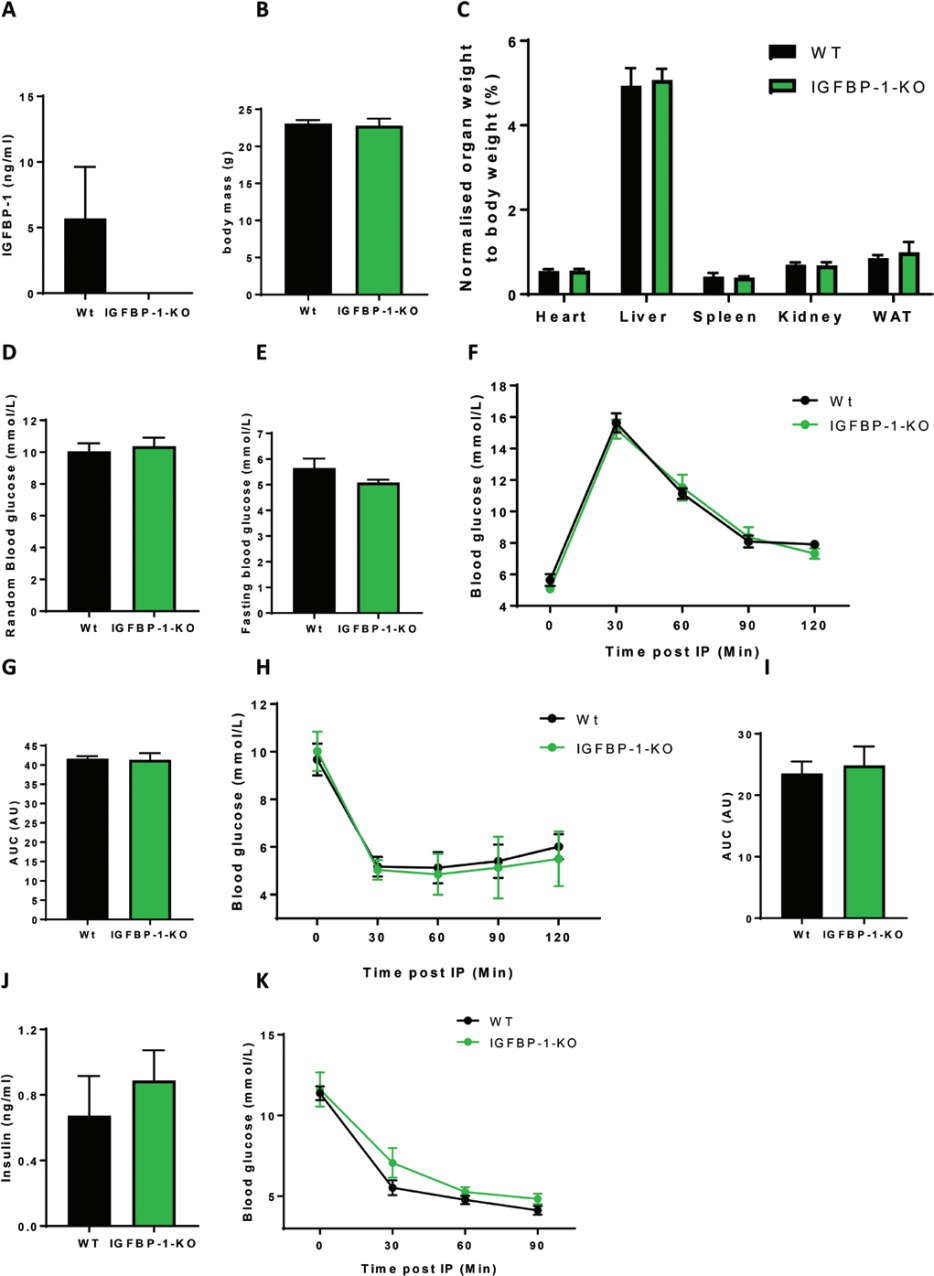
Metabolic profiling of IGFBP-1-KO mice. (A) Plasma was taken from IGFBP- 1-KO mice and wildtype litter mate controls, ELISA data confirmed IGFBP-1 knockdown (IGFBP-1-KO 0 ± 0 v Wt 5.69 ± 3.93 ng/mL). (B) Body mass was no different between groups (IGFBP-1-KO 22.8 ± 0.9 v Wt 23.1 g ± 0.9). (C) There was no difference in organ weights of IGFBP-1-KO mice when compared with wild-type litter mate control mice when normalized to body weight (Heart Wt 0.55 ± 0.04 v IGFBP-1-KO 0.56 ± 0.04) (Liver Wt 4.9 ± 0.4 v IGFBP-1-KO 5.07 ± 0.04) (Spleen Wt 0.4 ± 0.08 v IGFBP-1-KO 0.39 ± 0.03) (Kidney Wt 0.68 ± 0.05 v IGFBP-1-KO 0.6 ± 0.07) (WAT Wt 0.85 ± 0.07 v IGFBP-1-KO 0.9 ± 0.2). (D) Random blood glucose was no different between groups (IGFBP-1-KO 10.4 ± 0.5 v Wt 10 ± 0.5). (E) Fasting blood glucose was no different between groups (IGFBP-1-KO 5.1 ± 0.1 v Wt 5.6 ± 0.4). (F) Change in blood glucose levels after an IP injection of glucose, showed no difference in glucose handling, also shown by area under the curve analysis in **G** (IGFBP-1-KO 41.3 ± 1.8 v Wt 41.6 AU ± 0.7). (H) Change in blood glucose levels after an IP injection of insulin, showed no difference in insulin sensitivity, also shown in area under the curve analysis in **I:** (IGFBP-1-KO 24.8 ± 3.1 v Wt 23.5 AU ± 1.9). (J) Random plasma insulin was no different between groups (IGFBP-1-KO 0.89 ± 0.18 v Wt 0.67 ± 0.24). (K) Change in blood glucose levels after an IP injection of IGF-I shows that IGFBP-1-KO mice was not significantly different between the groups. Data are presented as mean ± SEM. N = 6 to 8 per group unless otherwise stated. (*P ≤ 0.05).

**Figure 2.**
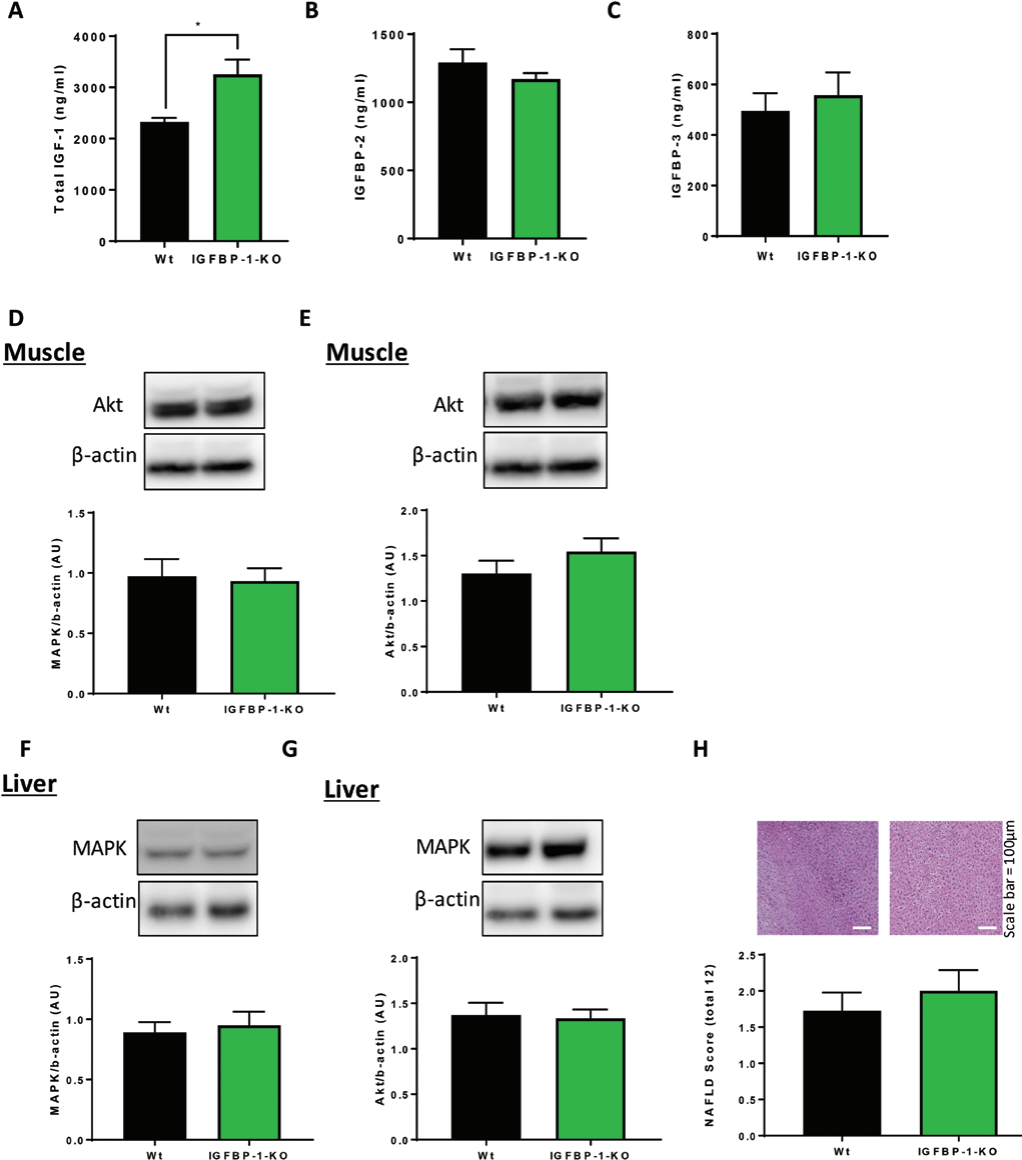
Plasma levels and signaling nodes in IGFBP-1 knockdown mice. (A) Plasma was taken from IGFBP-1-KO mice and wild-type litter mate controls and there was an increase in circulating total IGF-I levels (IGFBP-1-KO 3257 ± 285.8 V Wt 2327 ± 77.61 ng/mL). N = 5 to 7 per group for plasma samples. (B) Plasma was taken from IGFBP-1-KO mice and wildtype litter mate controls and there was no difference in circulating IGFBP-2 levels (IGFBP-1-KO 1171 ± 44.09 V Wt 1292 ± 97.36 ng/mL) C: Plasma was taken from IGFBP-1-KO mice and wild-type litter mate controls and there was no difference in circulating IGFBP-3 levels (IGFBP-1-KO 557.8 ± 90.03 V Wt 495.3 ± 70.1). (D,E) There was no difference in AKT or MAPK expression in the muscle of IGFBP-1-KO mice when compared with their wild-type litter mate controls. (F,G) There was no difference in AKT or MAPK expression in the liver of IGFBP-1-KO mice when compared to their wildtype litter mate controls. N = 5 per group for blotting studies. (H) Histological examination of liver showed there was no difference in non-alcoholic fatty liver disease (NAFLD) score (Wt 1.8 ± 0.2 V IGFBP-1-KO 2 ± 0.3) (n = 4). Data are presented as mean ±SEM. (*P ≤ .05).

To determine whether the presence of IGFBP-1 is essential for metabolic homeostasis and is implicated in physiological glucose regulation, we undertook detailed metabolic phenotyping in IGFBP-1-KO mice and their wild-type littermate controls. We first confirmed that IGFBP-1 was undetectable in the circulation of IGFBP-1-KO mice (Fig. 1A). IGFBP-1-KO mice had comparable body mass and wet organ weights to wild-type littermate controls (Fig. 1B and C). Glucose homeostasis was unchanged in IGFBP-1-KO mice, as shown by comparable random plasma glucose levels, fasting glucose levels, glucose tolerance test, and area under the curve analysis to controls (Fig. 1D-G). Insulin sensitivity and insulin concentrations were also the same (Fig. 1H-J). The hypoglycemic response to IGF-I administration was comparable in IGFBP-1-KO mice and wild-type littermate controls (Fig. 1K). Total circulating IGF-I concentration was significantly increased in IGFBP-1-KO mice (Fig. 2A), but circulating levels of IGFBP-2 and IGFBP-3 were not different from wild-type littermate controls (Fig. 2B and 2C). Expression of the metabolic signaling kinases AKT and MAPK in muscle and liver were no different between the genotypes (Fig. 2D–G). Accumulation of fat in the livers of IGFBP-1-KO mice, as shown by NAFLD scoring (Fig. 1H), was unchanged.

**Figure 3.**
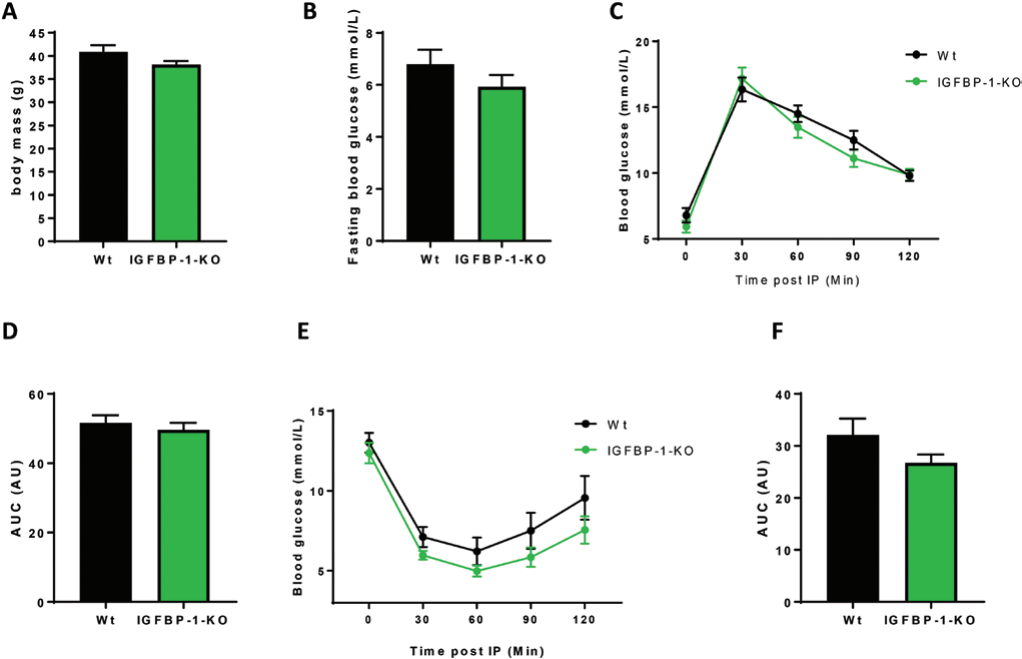
Metabolic profiling of IGFBP-1-KO obese mice. (A) Body mass was no different between groups (IGFBP-1-KO 38.8 ± 0.8 v Wt 40.8g ± 1.5). (B) Fasting blood glucose was no different between groups (IGFBP-1-KO 5.9 ± 0.44 v Wt 6.8 ± 0.55). (C) Change in blood glucose levels after an IP injection of glucose, showed no difference in glucose handling, also shown by area under the curve analysis in D (IGFBP-1-KO 49.6 ± 2 v Wt 51.6AU ± 2.2). (E) Change in blood glucose levels after an IP injection of insulin showed no difference in insulin sensitivity, also shown in area under the curve analysis in F (IGFBP-1-KO 26.76 ± 1.6 v Wt 32.11AU ± 3.1). Data are presented as mean ± SEM. N = 6 to 8 per group. (*P ≤ .05).

Having excluded an obligatory role for IGFBP-1 in maintaining normal metabolism, we next investigated whether loss of circulating IGFBP-1 predisposes to obesity-induced glucose intolerance and insulin resistance, as suggested by human observational data [9–11]. IGFBP-1-KO mice and their wild-type littermate controls were fed a 60% high-fat diet for 8 weeks before metabolic profiling was performed. IGFBP-1-KO mice had comparable body mass to wild-type littermate controls after 8 weeks of high-fat feeding (Fig. 3A). Glucose homeostasis after 8 weeks of high-fat feeding was no different in the IGFBP-1-KO mice, as shown by fasting glucose levels, glucose tolerance test, and area under the curve analysis (Fig. 3B–D respectively). Insulin sensitivity was also no different between the 2 genotypes (Fig. 3E and 3F).

### Vascular phenotype of IGFBP-1 knockout mice

**Figure 4.**
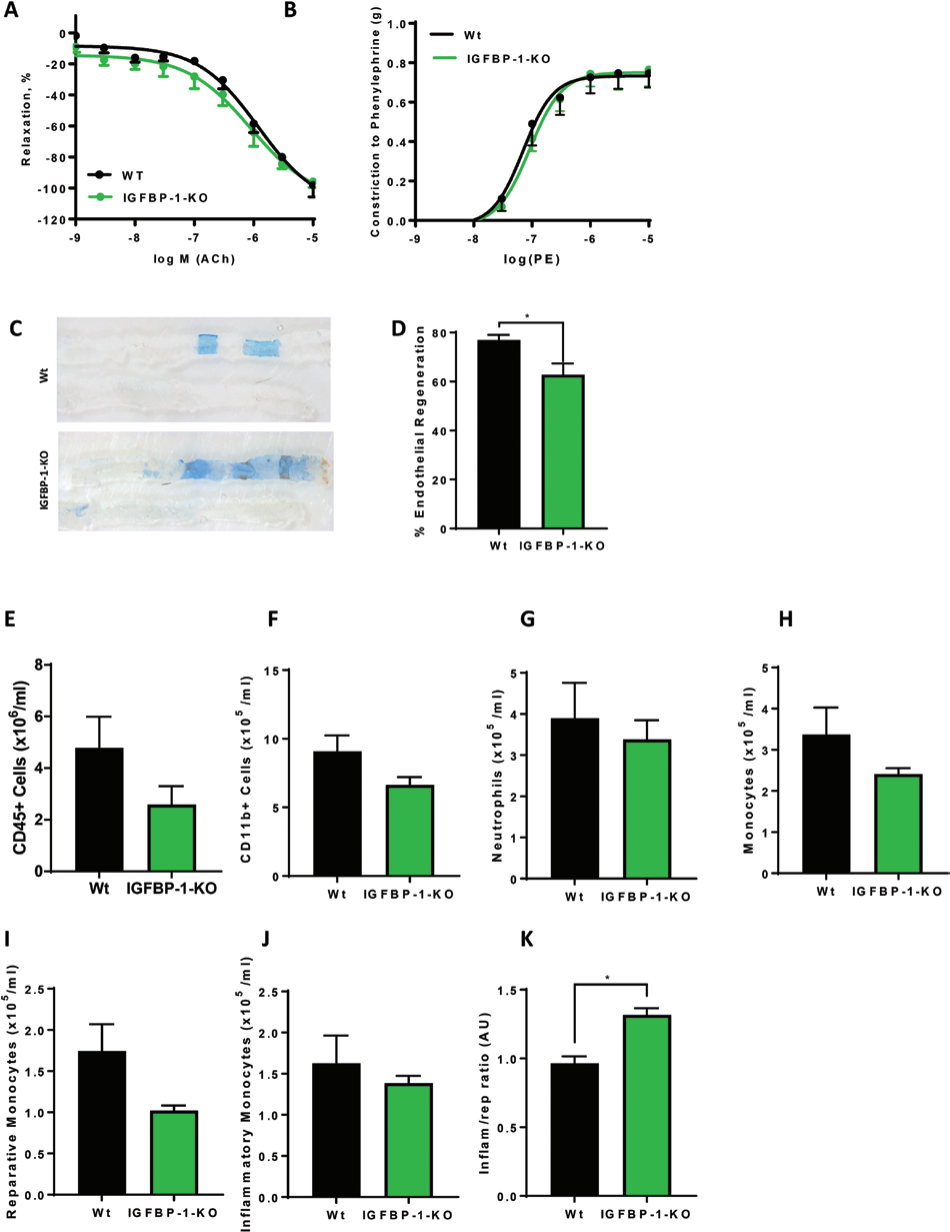
IGFBP-1 knockdown and endothelial function. (A) Relaxation responses to acetylcholine (Ach), expressed as percent reversal of phenylephrine induced contraction was the same between the genotypes. (B) Constrictor responses to PE was the same between the genotypes (N = 3–4). C: Representative in situ Evans blue staining 5 days after vascular injury (blue staining indicates denuded endothelium) in WT and IGFBP-1-KO mice (magnification ×20) (Sham bottom). (D) Endothelial regeneration 5 days after vascular injury is reduced in IGFBP-1-KO mice when compared to litter mate controls (IGFBP-1-KO 62.82 ± 4.5 V Wt 76.9 ± 2.1) (N = 4–6 per group). (E–J) There is no difference in CD45^+^ cells (IGFBP-1-KO 2.59 ± 0.7 V Wt 4.78 ± 1.2 x106/mL), CD11b+ cells (IGFBP-1-KO 6.6 ± 0.56 V Wt 9.08 ± 1.16 × 10^5^/mL), neutrophil numbers (IGFBP-1-KO 3.3 ± 0.46 V Wt 3.8 ± 0.86 x105/mL), monocyte cells (IGFBP-1-KO 2.4 ± 0.14 V Wt 3.37 ± 0.06 × 10^6^/mL), reparative monocytes (IGFBP-1-KO 1.023 ± 0.059 V Wt 1.75 ± 0.32 × 106/mL) or inflammatory monocytes (IGFBP-1-KO 1.38 ± 0.08 V Wt 1.62 ± 0.33 × 10^6^/mL) in IGFBP-1-KO mice when compared with controls. (K) There is a significant skewing of inflammatory to reparative monocytes IGFBP-1-KO mice when compared with controls (IGFBP-1-KO 1.32 ± 0.04 V Wt 0.96 ± 0.05) (n = 5). Data are presented as mean ± SEM. (*P ≤ .05).

We first examined whether IGFBP-1 is essential for normal vascular responses. Conduit artery vasomotor function in aortic rings ex vivo was comparable between IGFBP-1-KO mice and controls as shown by relaxation responses to acetylcholine (Ach) and constrictor responses to phenylephrine (PE) (Fig. 4A and 4B respectively). Having previously observed enhanced reparative capacity of the endothelium in IGFBP-1 overexpressing mice [16], we examined whether the presence of endogenous IGFBP-1 is necessary for effective regeneration of wire-injured endothelium. IGFBP-1-KO mice had significantly impaired endothelial regeneration 5 days after vascular injury compared with wild-type littermate controls (Fig. 4C and 4D). In recognition of the role of circulating inflammatory cells in vascular repair and the role of the IGF axis in immune modulation, we examined the effect of IGFBP-1 deletion on circulating immune cells. There was no difference in circulating numbers of CD45^+^ cells, CD11b^+^ myeloid cells, neutrophils, monocytes, reparative monocytes, or inflammatory monocytes between IGFBP-1-KO and wild-type littermate controls (Fig. 4E–J). However, there was a significant inflammatory monocyte skew in IGFBP-1-KO mice compared with wild-type littermate controls (Fig. 4K).

### Deletion or overexpression of IGFBP-1 does not modulate angiogenesis in healthy mice

**Figure 5.**
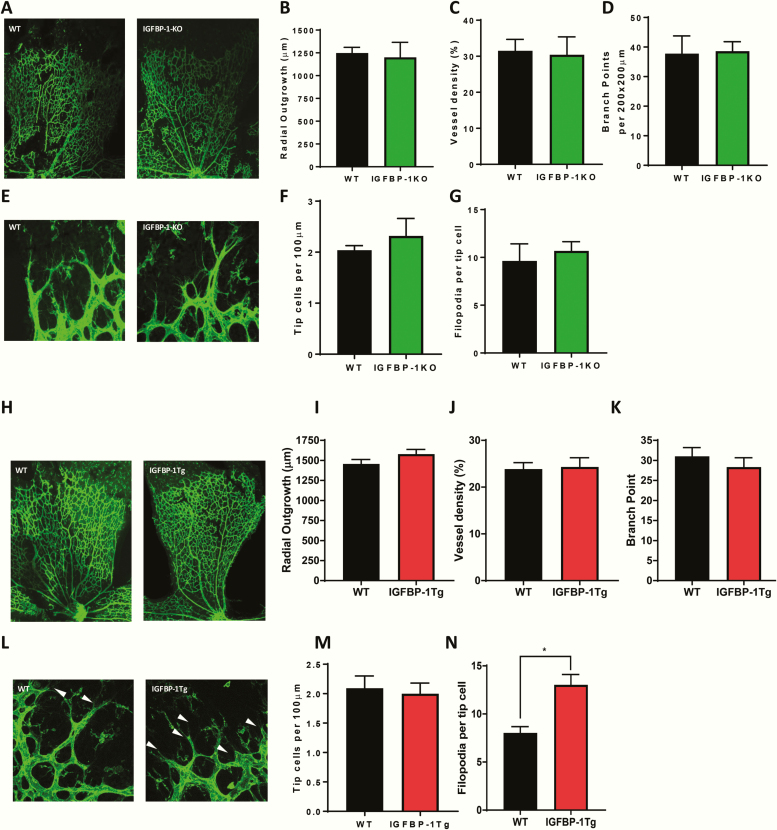
IGFBP-1 expression and developmental angiogenesis. (A–G) Retinas from IGFBP-1-KO mice p5 and wild-type litter mate controls were used to investigate the effects of IGFBP-1 knockout on developmental retinal angiogenesis. (A) Representative whole mount images. (B) There was no difference in vascular outgrowth (IGFBP-1-KO 1201 ± 165.4 V Wt 1248 ± 62.55), (C) vessel density (IGFBP-1-KO 30.4 ± 5 V Wt 31.5 ± 3.2), or (D) branching complexity (IGFBP-1-KO 38.6 ± 3.2 V Wt 37.8 ± 6) between IGFBP-1-KO mice and wild-type litter mate controls. (E) Representative images of vascular front of retinas from IGFBP-1-KO mice and wild-type litter mate controls. (F) There is no difference between tip cell number (IGFBP-1-KO 2.3 ± 0.34 V Wt 2.1 ± 0.1) or (G) number of filopodia (IGFBP-1-KO 10.7 ± 0.95 V Wt 9.3 ± 1. 78) between IGFBP-1-KO mice and wild-type litter mate controls (N = 4–5 per group). (H–N) Retinas from hIGFBP-1 overexpressing mice and wildtype litter mate controls were used to investigate the effects of hIGFBP-1 overexpression on retinal angiogenesis. (H) Representative whole mount images. (I) There is no difference in vascular outgrowth between IGFBP-1-tg mice and wildtype litter mate controls (tg 1579 ± 58.4 V Wt 1456 ± 55.6). (J) There is no difference in vessel density between IGFBP-1-tg mice and wild-type litter mate controls (tg 24.3 ± 2 V Wt 23.9 ± 1.4). (K) There is no difference in branching complexity between IGFBP-1-tg mice and wild-type litter mate controls (tg 28.4 ± 2.3 V Wt 31 ± 2.2). (L) Representative vascular front images. (M) There is no difference between tip cell number between IGFBP-1-tg mice and wild-type litter mate controls (tg 2 ± 0.18 V Wt 2.1 ± 0.21). (N: There is an increase in number of filopodia (white arrow head) in IGFBP-1-tg mice when compared with wild-type litter mate controls (tg 13 ± 1.1 V Wt 8 ± 0.66). N = 5 to 7 per group. Data are presented as mean ± SEM.

To investigate whether IGFBP-1 modulates physiological angiogenesis, we compared vascular architecture in the neonatal retina between p5 IGFBP-1-KO and IGFBP-1 overexpressing mice and their respective wild-type littermate controls. There was no difference in retinal vascular development between the IGFBP-1-KO and wild-type littermate controls (Fig. 5A–G). Similarly, there was no difference in retinal vascular development between IGFBP-1 overexpressing mice and wild-type littermate controls (Fig. 5H–M), although the number of filopodia per tip cell was higher in IGFBP-1 overexpressing mice (Fig. 5N).

**Figure 6.**
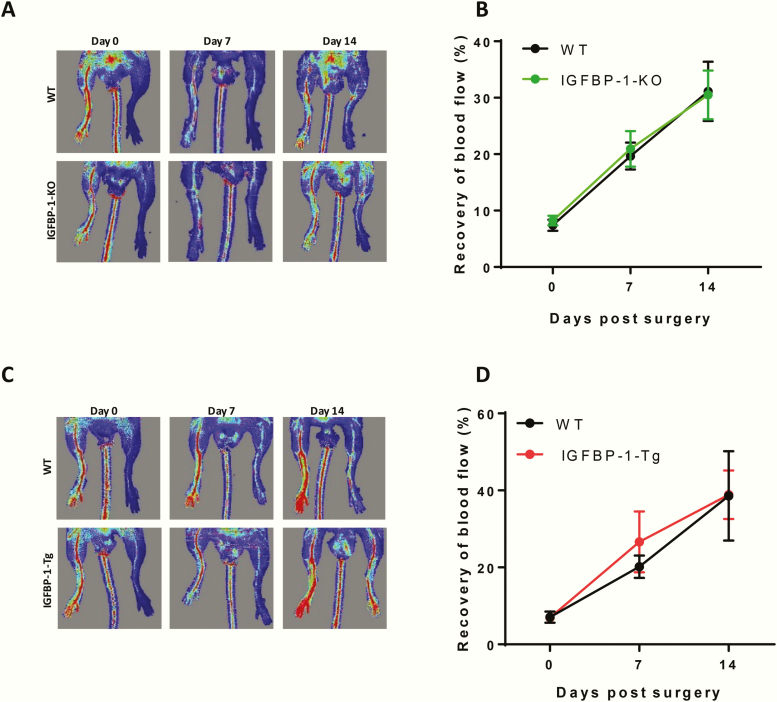
IGFBP-1 expression and pathological angiogenesis. (A,B) Mice with IGFBP-1 knock down were used to investigate recovery from hind limb ischemia as a model of pathological angiogenesis. (A) Representative laser Doppler perfusion images of mouse hind limbs on day 0, 7, and 14 after injury. (B) Quantitative analysis of the perfusion recovery measured by laser Doppler. The index was calculated as the ratio of ischemic to nonischemic hind limb perfusion. There is no difference in IGFBP-1-KO mice compared with wild-type litter mate controls There was no difference in necrotic toes between the genotypes (data not shown). N = 6 to 8 per group. (C,D) Mice overexpressing hIGFBP-1 were used to investigate recovery from hind limb ischemia. (C) Representative laser Doppler blood perfusion images of mouse hind limbs on day 0, 7, and 14 after injury. (D) Quantitative analysis of the perfusion recovery measured by laser Doppler. The index was calculated as the ratio of ischemic to non-ischemic hind limb blood perfusion. There was no difference in necrotic toes between the genotypes (data not shown) N = 7.

To investigate whether IGFBP-1 influences pathological angiogenesis in response to ischemia due to arterial occlusion, IGFBP-1-KO and IGFBP-1 overexpressing mice and their respective wild-type littermate controls underwent femoral artery ligation. There was no difference in recovery of hindlimb perfusion, as assessed by laser Doppler imaging, between IGFBP-1-KO and wild-type litter mate (Fig. 6A and 6B) or between IGFBP-1 overexpressing mice and their wild-type littermate controls (Fig. 6C and 6D).

### hIGFBP-1 overexpression improves recovery of perfusion following induction of hindlimb ischemia in insulin resistant mice

**Figure 7.**
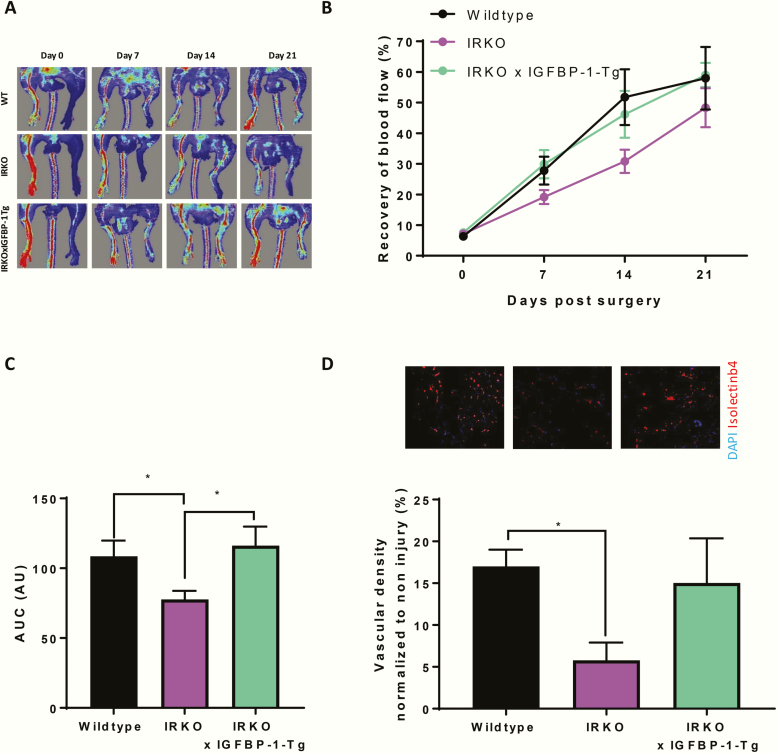
IGFBP-1 over expression in IRKO mice which have impaired hind limb ischemia recovery. (A–D) IRKO mice were used as a model of insulin resistance to investigate the effects on hind limb ischemia recovery. (A) Representative laser Doppler blood perfusion images of mouse hind limbs on day 0, 7, 14, and 21 after injury. (B) Quantitative analysis of the perfusion recovery measured by laser Doppler. The index was calculated as the ratio of ischemic to non-ischemic hind limb blood perfusion. There is significant impaired recovery in IRKO mice compared with wild-type litter mate controls at day 7 post surgery (IRKO 19.1% ± 2.3 V Wt 27.8% ± 4.6) and also shown in area under the curve in C (IRKO 77.8 ± 6.1 V Wt 108.6 ± 11 v IRKOxIGFBP-1-Tg 116.2 ± 13.7). IGFBP-1 overexpression improves recovery when compared to IRKO wild-type litter mate controls, as shown in area under the curve (IRKO 77.7 ± 6.1 V IGFBP-1 × IRKO 116.2 ± 13.7). (D) Gastrocnemius from ischemic and nonischemic limbs were stained with isolectin B4 (red) as a marker of endothelial cells and DAPI (blue) as a nuclear stain and capillary density quantified (Wt 17 ± 2 V IRKO 5.8 ± 2.1 V IRKO × IGFBP-1-Tg 15.03 ± 5.3) N = WT 6, IRKO 12, and IGFBP-1 × IRKO 19. Data are presented as mean ± SEM. (*P ≤ .05).

On the basis that a positive effect of IGFBP-1 on endothelial repair is only observed in insulin-resistant and not insulin-sensitive mice [16], we next investigated whether IGFBP-1 modulates angiogenesis in the setting of insulin resistance. We crossed IGFBP-1 overexpressing mice with mice hemizygous for knockout of the insulin receptor (IRKO) that are known to be insulin resistant in the vasculature [42]. We first confirmed that IRKO mice had impaired recovery from hindlimb ischemia compared with wild-type littermate controls (Fig. 7A–D). Overexpression of human IGFBP-1 in IRKO mice ameliorated the detrimental effect of insulin resistance on recovery from hindlimb ischemia and vascular density in ischemic muscle (Fig. 7A–D).

### rIGFBP-1 enhances endothelial cell angiogenic properties via IGF binding

**Figure 8.**
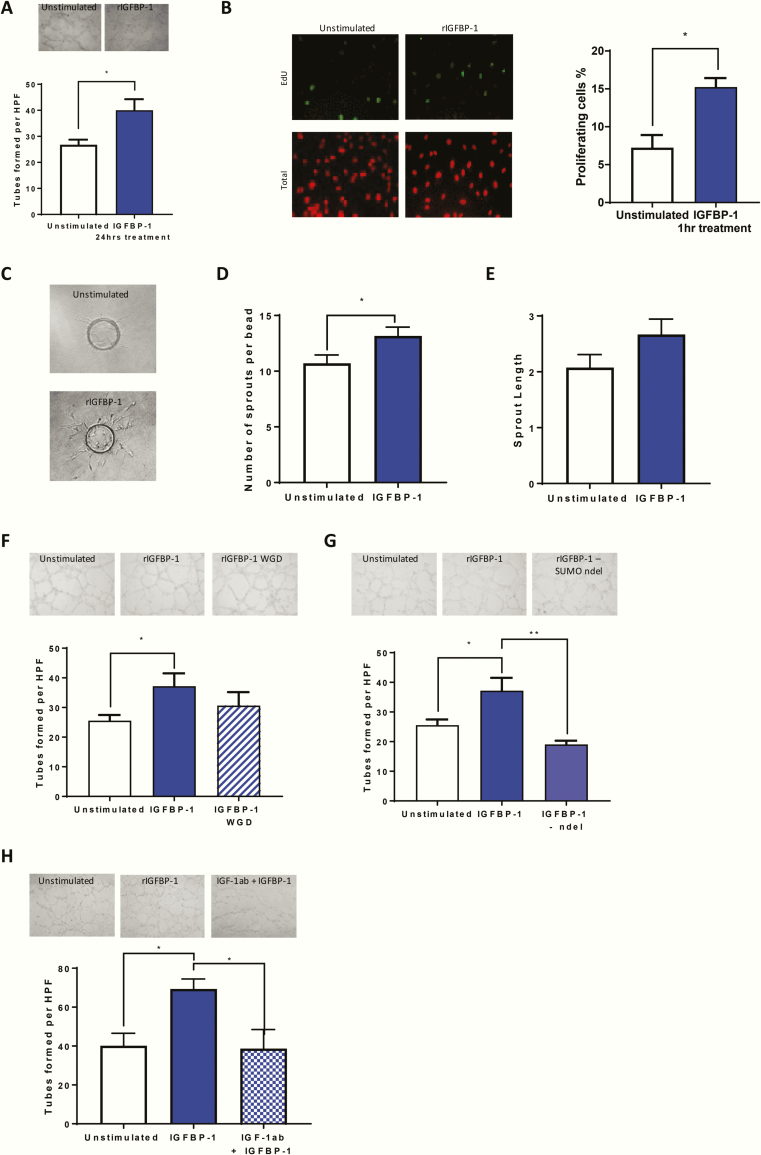
IGFBP-1 and angiogenic properties in vitro. (A) HUVECs were used to investigate the effects of rIGFBP-1 on endothelial cell tube formation on Matrigel. Treatment with rIGFBP-1 for 24 hours before being seeded onto Matrigel enhanced endothelial cell tube formation when compared to unstimulated cells (IGFBP-1 40 ± 4.26 V UN 26.75 ± 1.98). (B) HUVECs were used to investigate the effects of rIGFBP-1 on endothelial cell proliferation using an EDU incorporation assay. Treatment with rIGFBP-1 for 1hr before EdU was added enhanced endothelial cell proliferation when compared to unstimulated cells (IGFBP-1 15.24 ± 1.8 V UN 7.23 ± 1.67). (C) HUVECs were used to investigate the effects of rIGFBP-1 on endothelial cell sprouting using a cytodex bead assay. Representative images are shown. Beads were either grown in the presence of IGFBP-1 or not. (D) Beads grown in the presence of IGFBP-1 had a significantly increased number of sprouts (IGFBP-1 13.2 ± 0.79 V UN 10.7 ± 0.75). (E) Beads grown in the presence of IGFBP-1 had a trend for enhanced sprout length (IGFBP-1 2.7 ± 0.28 V UN 2.1 ± 0.23). (F) HUVECs were used to investigate the effects of mutant IGFBP-1 on endothelial cell tube formation on Matrigel. Treatment with WGD mutant IGFBP-1 for 24 hours before being seeded onto Matrigel had no effect on endothelial cell tube formation when compared with IGFBP-1 treated cells (IGFBP-1 37.2 ± 4.3 V WGD 30.6 ± 4.6). G: Treatment with amino-terminal deletion mutant IGFBP-1 for 24 hours before being seeded onto Matrigel reduced endothelial cell tube formation when compared with IGFBP-1 cells (IGFBP-1 37.18 ± 4. V NDEL 19.05 ± 1.3). (H) Treatment with an IGF-I neutralizing antibody and IGFBP-1 for 24 hours before being seeded onto Matrigel reduced endothelial cell tube formation when compared to IGFBP-1 only treated cells (IGFBP-1 69.3 ± 5.1. V IGF-1ab + IGFBP-1 38.7 ± 9.8). Data are presented as mean ± SEM. (*P ≤ .05; **P ≤ .01). N = 4 per group.

To explore potential mechanisms by which IGFBP-1 positively modulates angiogenesis, we used HUVECs to investigate the proangiogenic effect of rIGFBP-1 in vitro. We employed Matrigel tube formation assays to mimic in vivo endothelial cell capillary formation. Incubation with rIGFBP-1 (500 ng/mL 24 hours) enhanced endothelial tube forming in HUVECs (Fig. 8A). To probe potential mechanisms behind IGFBP-1 enhanced tube formation we investigated the effect of rIGFBP-1 on functional properties of endothelial cells. We used an 5-ethynyl-2’-deoxyuridine (EdU) incorporation assay to probe whether IGFBP-1 enhances proliferation. After incubation with rIGFBP-1 for only 1 hour, endothelial cell proliferation was significantly enhanced (Fig. 8B). We then used a cytodex bead assay to determine whether IGFBP-1 increases endothelial sprouting. HUVEC-coated beads grown in the presence of IGFBP-1 displayed a significantly increased number of sprouts and a trend for enhanced sprout length (Fig. 8C–E). We used site-directed mutagenesis and deletion to identify the structural motifs of rIGFBP-1 responsible for the proangiogenic effects. We first mutated the RGD domain to WGD to prevent interaction of rIGFBP-1 with cell surface integrin receptors. We also deleted the amino-terminal domain of rIGFBP-1 to prevent binding of IGFs. We observed enhancement of tube formation following pre-incubation with rIGFBP-1. There was a trend to increased tube formation compared with control despite WGD mutation, and the increase in tube formation was not significantly less than that seen with rIGFBP-1 stimulation, raising the possibility that the RGD domain is not involved in the angiogenesis effect seen. More work is needed however to fully understand the role of this domain (Fig. 8F). However, deletion of the N-terminus of IGFBP-1 (IGFBP-1 (ΔIGF-I)), which prevented IGF-I binding [43], obliterated its proangiogenic action (Fig. 8G). An essential role for IGF-I in the modulation of angiogenesis by IGFBP-1 was confirmed by repeating these experiments with an IGF-I neutralizing antibody (Fig. 8H).

## Discussion

In this report, we employed a loss of function approach to determine whether IGFBP-1 has an obligatory role in metabolic and vascular homeostasis. We demonstrate that deletion of IGFBP-1 does not modulate metabolic phenotype in lean or obese mice, drawing into question the argument that low levels of IGFBP-1 are permissive for the development of diabetes in humans. In the vasculature, we similarly demonstrated that deletion of IGFBP-1 does not affect conduit artery vasomotor responses. In contrast, the capacity for regeneration of injured vascular endothelium was significantly blunted in IGFBP-1-KO mice, indicating that IGFBP-1 does contribute to endogenous vascular repair. In animals with intact insulin signaling, neither deletion nor overexpression of IGFBP-1 modulates angiogenesis. However, in insulin-resistant mice increasing IGFBP-1 does positively influence recovery of perfusion following induction of hindlimb ischemia. At the cellular level, IGFBP-1 stimulated endothelial proliferation and sprouting though a molecular mechanism dependent on IGF-I but not RGD ligation.

Clinical data from diverse populations suggest that low levels of IGFBP-1 may be permissive for the development of type 2 diabetes in humans [6, 7, 10, 11]. In a population-based study of individuals of European or Pakistan origin, IGFBP-1 was independently associated with impaired 2-hour glucose tolerance and every 2.7 ng/mL increase in IGFBP-1 was associated with a 40% risk reduction for developing impaired glucose tolerance [6]. A similar population study in healthy pubertal children, identified a strong positive correlation between IGFBP-1 and insulin sensitivity and an inverse correlation with body mass [7]. In a prospective study with 17 years of follow-up, low IGFBP-1 levels strongly predicted the long-term development of type 2 diabetes [11]. Although persuasive in supporting the argument that low levels of IGFBP-1 serve as a driver for the development of metabolic disorders, such studies cannot prove a causal role for IGFBP-1. We previously reported that increased levels of IGFBP-1 promote insulin sensitivity and improve glucose tolerance [15, 17]. These findings indicate that increasing IGFBP-1 may be useful as a therapeutic strategy, but do not answer the question of whether IGFBP-1 plays an obligatory part in normal physiology and whether its loss is harmful.

To address whether IGFBP-1 has an essential role in metabolic and vascular physiology, we extended our previous “gain-of-function” studies by performing “‘loss-of-function” investigations reported here. We found that deletion of IGFBP-1 did not materially affect metabolic phenotype in mice. Results of glucose tolerance, insulin tolerance, and IGF-I tolerance tests were similar in mice lacking IGFBP-1. Failure of IGFBP-1 deletion to alter metabolic phenotype suggests that IGFBP-1 is not essential for normal metabolic physiology, and that loss of IGFBP-1 activity is not causally implicated in susceptibility to diabetes of humans with low IGFBP-1 levels. Instead, IGFBP-1 may be acting as a biomarker for insulin sensitivity as previously suggested [7]. However, there are some caveats to this interpretation. Genetic deletion studies in knockout animals may be confounded by upregulation of other bioactive molecules in compensatory or adaptive responses. Plasma levels of IGFBP-2 and IGFBP-3 were comparable between IGFBP-1-KO mice and their wild-type littermate controls, implying that the IGFBP-1 knockdown did not result in a compensatory increase in either of these IGFBPs which are known to be implicated in glucose regulation [44–48]. However, we cannot exclude altered levels of other bioactive molecules. We did find, consistent with previous reports, that IGFBP-1-KO mice had higher total IGF-I levels [49]. As IGF-I promotes insulin sensitivity [50], this increase in circulating IGF-I may compensate for the lack of IGFBP-1 and explain why IGFBP-1-KO do not display impaired glucose tolerance or insulin resistance in line with population data in the literature. It is also worth noting that mouse physiology does not completely reflect the human situation and this could be a possible explanation for the lack of effect of IGFBP-1 deletion on metabolic parameters. However, our results are consistent with the findings of a prospective study of 615 normoglycemic men and women [10], in which total IGF-I concentrations were inversely associated with subsequent 2-hour glucose concentrations, but only in people with low concentrations of IGFBP-1, suggesting that the biological interaction between IGF-I and IGFBP-1 is important in glucose homoeostasis.

Several studies have shown that IGFBP-1 levels are correlated with BMI, raising the possibility that IGFBP-1 is implicated in obesity [3, 6–8]. To investigate if loss of IGFBP-1 predisposes to obesity-related glucose intolerance and insulin resistance, we challenged IGFBP-1-KO mice and their wild-type littermate controls with a 60% high-fat diet for 8 weeks. After high-fat feeding, IGFBP-1-KO mice had comparable body mass, glucose homeostasis, and insulin sensitivity, indicating that IGFBP-1 is not causally implicated in protection from obesity and obesity-impaired glucose tolerance.

We next investigated if loss of IGFBP-1 influences vascular function, recognizing that several studies report a negative correlation between IGFBP-1 and biomarkers of cardiovascular disease such as blood pressure, BMI, waist/hip ratio, and fasting insulin levels [6–8]. Furthermore, in a prospective study, it was found that low baseline levels of IGF-I and IGFBP-1 increased the risk of fatal ischemic heart disease among both elderly men and women independent of prevalent ischemic heart disease and other cardiovascular risk factors [12]. In contrast to the positive influence of IGFBP-1 on vascular phenotype, we reported in gain-of-function studies previously, in which IGFBP-1 increased endothelial nitric oxide generation [15], in the current dataset aortic vasomotor function was not altered by deletion of IGFBP-1. This implies that IGFBP-1 does not play a critical role in maintenance of normal vasomotor physiology. However, it should be recognized that both IGFBP-1 and IGF-I stimulate nitric oxide generation and have been shown to reduce atherosclerosis in mice [15, 26]. We speculate that in relation to endothelial function, increased IGF-I is compensating for loss of IGFBP-1 in IGFBP-1-KO mice.

IGFBP-1 is required for the endothelium to respond appropriately to injury. Following wire injury to the femoral artery, recovery of endothelial coverage was significantly impaired in IGFBP-1-KO compared with wild-type controls. As discussed further below, we found that IGFBP-1 significantly upregulates endothelial cell proliferation, the loss of which may contribute to deficient repair in knockout animals. Additionally, endothelial regeneration is influenced by cell types other than endothelial cells, not least circulating inflammatory cells [51]. Although total abundance of monocytes was unaltered by IGFBP-1 deletion, we observed that the ratio of inflammatory to reparative monocytes was adversely skewed in IGFBP-1-KO mice. Further work is required to determine whether adverse monocyte skewing predisposes IGFBP-1-KO to impaired endothelial regeneration [52]. Diminished capacity for endothelium to regenerate in response to mechanical or biochemical injury contributes to adverse vascular remodeling and atherosclerosis [53]. Loss of this capacity in individuals with low IGFBP-1 levels may therefore be implicated in increased vascular risk and predisposition to macrovascular disease [13, 54].

Angiogenesis is a tightly regulated process that is vital during postnatal development and contributes to several pathologies including cancer and response to ischemia arising from occlusive vascular disease. Members of the IGFBP family have been ascribed positive and negative roles in angiogenesis which are actioned both through their binding to IGFs and through IGF-independent mechanisms [19]. Although modulatory effects on angiogenesis have been well documented for some of the IGFBPs, little is known about the angiogenic properties of IGFBP-1. We addressed this by carrying out loss-of-function and gain-of-function studies in vivo. Vascular patterning in the postnatal retina remained intact in IGFBP-1-KO mice, indicating that IGFBP-1 is not essential in developmental angiogenesis. Because the cellular processes and microenvironmental cues implicit in endothelial regeneration and sprouting angiogenesis differ, it is not surprising that the effect of IGFBP-1 on these readouts may diverge. Similarly, retinal vascular architecture was not materially affected by IGFBP-1 overexpression, although increased numbers of filopodia were observed in tips cells of IGFBP-1 overexpressing mice. Filopodia are thought to drive directional migration of tip cells in response to microenvironmental cues [55]; however, the relevance of increased numbers of filopodia in IGFBP-1-tg retinas in the absence of altered radial outgrowth is uncertain.

Neovascularization in response to ischemia in adult animals contributes to tissue perfusion in occlusive vascular disorders and has been targeted for exploitation clinically in therapeutic angiogenesis [19]. In mice with intact insulin signaling, neither IGFBP-1 knockout nor overexpression altered recovery of tissue perfusion after induction of hindlimb ischemia. This suggests that IGFBP-1 does not possess an obligatory role in pathological angiogenesis and increasing circulating IGFBP-1 is unable to augment the response to ischemia, at least in “metabolically normal” animals. However, angiogenic responses in young metabolically healthy animals are likely to be optimal with limited opportunity to be augmented further. To investigate whether elevated IGFBP-1 levels could promote recovery from hindlimb ischemia in a “disease” model, we crossed IGFBP-1 overexpressing mice with IRKO mice, which are hemizygous for knockout of the insulin receptor. IRKO mice display several phenotypic properties consistent with prediabetes in humans, including mild insulin resistance, endothelial dysfunction, and elevated blood pressure, and have impaired regeneration following arterial injury and impaired recovery from hindlimb ischemia [42, 56, 57]. In contrast to mice with intact insulin signaling, overexpression of IGFBP-1 in IRKO mice restored limb perfusion after induction of ischemia to normal levels observed in wild-type mice. This implies that IGFBP-1 can promote neovascularization in insulin-resistant animals but not in healthy animals with normal metabolism. We observed that IGFBP-1 has direct proangiogenic effects on endothelial cells in vitro, stimulating proliferation, sprouting, and tube formation. Given that insulin resistance was not modelled in vitro for these experiments, we deduce that IGFBP-1 is an inherently proangiogenic protein which lacks capacity to promote neovascularization in healthy animals but has angiogenic properties which are unmasked when animals are challenged by insulin resistance.

Our results provide novel insights into the molecular mechanisms by which IGFBP-1 stimulates angiogenesis and indicate that these are not IGF independent. Intriguingly, we discovered that ablation of the ability of IGFBP-1 to interact with cell surface integrins by site-directed mutagenesis of the RGD domain did not significantly impair its capacity to stimulate endothelial tube formation. This contrasts with the mechanisms by which IGFBP-1 stimulates nitric oxide generation in endothelium or increases insulin signaling in skeletal muscle, both of which are RGD domain dependent [16, 17]. Instead, upregulation of angiogenesis by IGFBP-1 was abrogated by preventing IGF-I binding or by inhibiting IGF-I with a blocking antibody. The mechanisms by which IGFBP-1 and IGF-I cooperate to promote angiogenic responses in endothelium require further investigation.

## Conclusions

In summary, the loss of function studies in IGFBP-1-KO mice reported here suggest that IGFBP-1 does not play an essential role in metabolic or vascular physiology. This draws into question the concept that low levels of IGFBP-1 are permissive for the development of cardiometabolic disease in humans. An important caveat is that total IGF-I levels were increased in IGFBP-1-KO mice, which prevents us from modelling the influence of low IGF-I and low IGFBP-1 in combination that is known to be associated with glucose intolerance in humans. Interestingly, IGFBP-1 was required for normal regeneration of endothelium, raising the possibility that IGFBP-1 deficiency may expose humans to vascular disease by reducing capacity for the endothelium to repair in response to injury. Finally, IGFBP-1 displays promise for therapeutic angiogenesis, as a pro-angiogenic protein with ability to increase perfusion in tissue ischemia in the setting of insulin resistance.

## Abbreviations

Ach: acetylcholine
BMI: body mass index
DPBS: Dulbecco’s phosphate-buffered saline
EDTA: ethylenediamine tetra-acetate
HUVEC: human umbilical vein endothelial cell
IGF: insulin-like growth factor
IGFBP: insulin-like growth factor binding protein
IRKO: knockout of the insulin receptor
NAFLD: nonalcoholic fatty liver disease
PE: phenylephrine

## Notes

***Author Contributions:*** N.J.H. designed and performed the in vitro and in vivo experiments, analyzed data, and wrote the manuscript. T.S./N.W./C.M./M.D. performed ex vivo studies. K.I.B./N.Y.Y. performed the in vivo studies. R.M.C./M.T.K./S.B.W. conceived the study, obtained funding, designed experiments, supervised the project and reviewed the manuscript. S.B.W. is the guarantor of the work and, as such, had full access to all the data in the study and takes responsibility for the integrity of the data and the accuracy of the data analysis.

## Additional Information

***Disclosure Summary***: I certify that neither I nor my co-authors have a conflict of interest as described above that is relevant to the subject matter or materials included in this Work.
